# Regional nigral neuromelanin degeneration in asymptomatic leucine-rich repeat kinase 2 gene carrier using MRI

**DOI:** 10.1038/s41598-024-59074-8

**Published:** 2024-05-09

**Authors:** Linlin Gao, Rahul Gaurav, Pia Ziegner, Jinghong Ma, Junyan Sun, Jie Chen, Jiliang Fang, Yangyang Fan, Yan Bao, Dongling Zhang, Piu Chan, Qi Yang, Zhaoyang Fan, Stéphane Lehéricy, Tao Wu

**Affiliations:** 1 Department of General Practice, Tianjin Union Medical Center, Tianjin, China; 2 Paris Brain Institute – ICM, INSERM U1127, CNRS UMR 7225, Pitié-Salpêtrière Hospital, Sorbonne Université, Paris, France; 3 Movement Investigations and Therapeutics Team (MOV’IT), Paris Brain Institute – ICM, Paris, France; 4 Center for NeuroImaging Research (CENIR), Paris Brain Institute – ICM, Hôpital Pitié-Salpêtrière, 47 Boulevard de l’Hôpital, 75013 Paris, France; 5 Department of Neurology (H.J.), University Hospital of Heidelberg, Heidelberg, Germany; 6 Department of Neurology, Xuanwu Hospital of Capital Medical University, Beijing, China; 7 Department of Neurology, Center for Movement Disorders, Beijing Tiantan Hospital, Capital Medical University, Beijing, China; 8 China National Clinical Research Center for Neurological Diseases, Beijing, China; 9 Department of Radiology, Guang’anmen Hospital, China Academy of Chinese Medical Sciences, Beijing, China; 10 Department of Radiology, Xuanwu Hospital of Capital Medical University, Beijing, China; 11 Department of Radiology, Keck School of Medicine, University of Southern California, Los Angeles, CA 90033 USA; 12 Department of Neuroradiology, Pitié-Salpêtrière Hospital, AP-HP, Paris, France

**Keywords:** Asymptomatic leucine-rich repeat kinase 2 (LRRK2) gene carrier, Parkinson’s disease (PD), MRI, Neuromelanin, Substantia nigra (SN), Computational neuroscience, Diseases of the nervous system, Neuroscience, Biomarkers, Neurology

## Abstract

Asymptomatic Leucine-Rich Repeat Kinase 2 Gene (LRRK2) carriers are at risk for developing Parkinson's disease (PD). We studied presymptomatic substantia nigra pars compacta (SNc) regional neurodegeneration in asymptomatic LRRK2 carriers compared to idiopathic PD patients using neuromelanin-sensitive MRI technique (NM-MRI). Fifteen asymptomatic LRRK2 carriers, 22 idiopathic PD patients, and 30 healthy controls (HCs) were scanned using NM-MRI. We computed volume and contrast-to-noise ratio (CNR) derived from the whole SNc and the sensorimotor, associative, and limbic SNc regions. An analysis of covariance was performed to explore the differences of whole and regional NM-MRI values among the groups while controlling the effect of age and sex. In whole SNc, LRRK2 had significantly lower CNR than HCs but non-significantly higher volume and CNR than PD patients, and PD patients significantly lower volume and CNR compared to HCs. Inside SNc regions, there were significant group effects for CNR in all regions and for volumes in the associative region, with a trend in the sensorimotor region but no significant changes in the limbic region. PD had reduced volume and CNR in all regions compared to HCs. Asymptomatic LRRK2 carriers showed globally decreased SNc volume and CNR suggesting early nigral neurodegeneration in these subjects at risk of developing PD.

## Introduction

Parkinson’s disease (PD) is a neurodegenerative movement disorder characterized by the depletion of dopaminergic neurons in the substantia nigra pars compacta (SNc)⁠^1^. The SNc neurodegeneration begins before the onset of PD during the prodromal stage of the disease^2–4^. This prodromal phase can be studied in subjects at-risk such as isolated rapid eye movement sleep behavior disorder (iRBD) and asymptomatic PD-related mutation carriers. Mutations in leucine-rich repeat kinase 2 gene (LRRK2) are the most commonly known cause of inherited PD^5,6^. LRRK2 mutations are found in different ethnic groups^7–10^. Asymptomatic LRRK2 mutation carriers, found in 3–4% of Chinese individuals, increases the risk of PD by approximately two-fold^11^. PD patients with LRRK2 mutation have Lewy body pathology and they show clinical signs similar to patients with idiopathic PD. Previous imaging studies have shown that asymptomatic LRRK2 carriers have reduced F-6-fluoro-L-dopa (FDOPA) uptake and dopamine transporter (DAT) binding^12,13^, greater SN hyperechogenicity^14,15^, and increased iron deposition in the SN^16^. These findings indicate that the nigrostriatal changes are present in asymptomatic LRRK2 carriers. However, regional nigral neurodegeneration in asymptomatic LRRK2 carriers remains unclear.

Neuromelanin (NM) sensitive MRI (NM-MRI) technique has been used to study the degeneration of dopaminergic neurons in the SNc and the locus coeruleus/subcoeruleus complex (LC/LsC). NM-iron compound has paramagnetic properties and presents a high signal intensity in high-resolution fast spin echo T1-weighted imaging^17^ and gradient echo with magnetization transfer preparation prepulse sequence^18^. NM-MRI can measure the NM-generated signals from the SNc and LC/LsC^19,20^. NM-MRI demonstrates a reduction in volume and contrast to noise ratio (CNR) in the SNc with high diagnostic accuracy in PD patients^21–24^ in early^24–27^ as well as in the prodromal phase^28,29^. Previous NM-MRI study suggested that the nigral degeneration pattern was similar in LRRK2-associated PD and idiopathic PD patients^30^.

The aim of the current study was to use NM-MRI technique to understand the global and regional changes inside the SNc using volume and signal intensities in asymptomatic LRRK2 carriers compared with idiopathic PD patients and healthy controls (HCs). This study would help us understand the characteristics of nigral NM neurodegeneration at the prodromal stage of PD.

## Methods

### Subjects

From April 2018 to June 2019, 15 asymptomatic LRRK2 carriers (including 8 G2385R mutation carriers and 7 R1628P mutation carriers) and 30 HCs from the community cohorts of the Beijing Longitudinal Study on Aging were enrolled in our study. Each asymptomatic LRRK2 carriers were matched individually to two HCs in order to match for age and sex. We also recruited 22 idiopathic PD patients from the Movement Disorders Clinic of the Xuanwu Hospital of Capital Medical University, Beijing, China. PD patients were diagnosed by two movement disorder specialists (Drs. J.M. and P.C.) according to the Movement Disorder Society (MDS) clinical diagnostic criteria for PD. Additionally, the criteria for asymptomatic LRRK2 carriers were verified by neurologists in the absence of MDS criteria for PD or related disorders. However, the LRRK2 carriers manifesting parkinsonism symptoms were not included in this study. The subjects with other neurological diseases or contraindications to MRI were excluded. This experiment was performed in accordance with the Declaration of Helsinki and was approved by the Institutional Review Board of Xuanwu Hospital. All subjects provided written informed consent prior to the experiment.

All participants were evaluated using the MDS Unified Parkinson’s Disease Rating Scale (MDS-UPDRS) off their anti-parkinsonian medication and the Hoehn & Yahr (H&Y) scale.

### MRI data acquisition

MRI data were acquired using a 3.0 Tesla scanner (Magnetom Skyra, Siemens, Germany) with a 20-channel receiver head and neck joint coil. A two-dimensional (2D) gradient echo (GRE) sequence with magnetization transfer contrast (MTC) was used to acquire NM-MRI with the following parameters: TR/TE, 180/2.5 ms; flip angle 25°; field of view 202 × 168 mm^2^; spatial resolution 0.35 × 0.35 mm^2^ (interpolated from 0.7 mm); slice thickness: 3 mm; 7 slices; measurement 7; scan time 3 min 48 s. The 7 measurements were co-registered and averaged to generate one image set. The orientation of the axial sections was set parallel to the anterior commissure posterior commissure line with coverage from the posterior commissure to the pons. FLAIR, T1 and T2-weighted images were obtained to exclude other neurological disorders or lesions. In addition, whole-brain sagittal three-dimensional (3D) T1-weighted magnetization-prepared rapid gradient-echo (MP-RAGE) imaging was acquired with the following parameters: TR/TE, 2530/2.98 ms; slice thickness 1 mm, flip angle 7°, display field of view (DFOV) 256 × 256 × 192 pixels, voxel size 1 × 1 × 1 mm^3^, scanning time 5 min 13 s.

### Image analysis

All image processing and analysis were performed using software programs written using in-house algorithm in MATLAB (The MathWorks Inc., MA, USA, vR2017b) combined with Statistical Parametric Mapping (SPM12, UK), FreeSurfer (MGH, USA, v5.3.0), FSL (FMRIB, UK, v5.0) and NiftyReg (v1.5.58).

#### Region of interest (ROI) selection

##### Whole SNc

**Figure 1:**
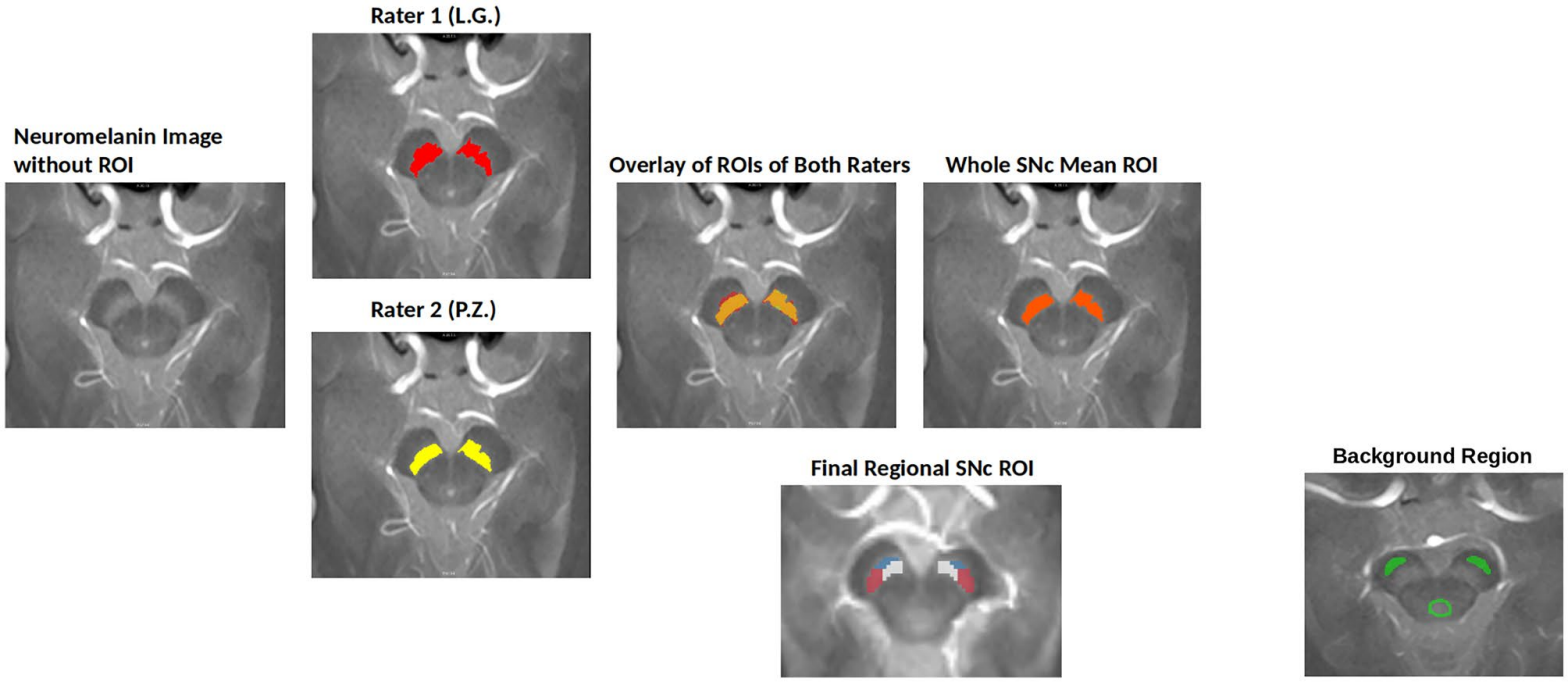
Illustration of whole and regional substantia nigra pars compacta (SNc) segmentation along with background region segmentation.

The whole SNc ROI segmentation was performed using the FreeSurfer viewer similar to previous studies (Fig. 1)^24,31^. SNc contours were delineated manually by two trained raters (rater 1: L.G., rater 2: P.Z.) around the area of high signal intensity as the hyperintense area dorsal to the cerebral peduncle and ventral to the red nucleus. The contours were continuous without any non-contiguous voxels. We segmented the three lowest slices of the visible SNc for each subject. The raters were blind to the clinical status of the participant.

Both raters also manually traced a background region (Fig. 1) that included the tegmentum and cerebral peduncles. Each subject’s SNc ROI and background ROI was segmented twice with a 4-week interval by the raters. Dice similarity coefficient and intraclass correlation coefficient (ICC) were calculated. A Dice and ICC of 0.81 to 1.00 was considered to be excellent and 0.61 to 0.80 to be good agreement. Lastly, for each participant, we applied fslmaths function of FSL to obtain a mean ROI between the first and second rater, which was used for further statistical analyses (Fig. 1).

##### Regional SNc

Briefly, NM images were aligned to an available study-specific average brain template obtained using another ongoing cohort study (ICEBERG, ClinicalTrials.gov: NCT02305147) as they have been tested in other previous studies^31,32^. Altogether, 114 subjects (38 each in HCs, prodromal parkinsonism and idiopathic early-stage PD patients group) were used to develop this template.

Thereafter, the regional masks were delineated on this template by experts based on earlier studies on nigral functional territories in non human primates in form of three territories inside the SN as dorsolateral sensorimotor, dorsomedial limbic and ventral associative^33^.

Henceforth, for obtaining regional masks for each subject, the NM image was rigidly co-registered to the corresponding 3D T1-weighted image. The 3D T1-weighted image was then aligned to the average brain template. The resulting transformation was applied to the NM image and the mean SNc ROI obtained between the first and second rater (described above in the Whole SNc ROI selection subsection). As final regional ROIs for each subject used in statistical analyses, we considered the intersections between the mean whole SNc ROI obtained using both raters and the three regional masks by applying fslmaths function of FSL.

#### Quantitative analysis

##### Whole SNc

The volumes of the manually obtained whole SNc ROIs were calculated using in-house MATLAB algorithm by employing fslstats function of FSL as the number of voxels in the ROIs of the three lowest contiguous image slices where the SNc was visible, multiplied by the voxel size. Total intracranial volume (TIV) was also obtained as a summation of gray matter, white matter and cerebrospinal fluid using the Computational Anatomy Toolbox (CAT12.1) for the Statistical Parametric Mapping software for MATLAB (SPM12). In order to normalize for the head size, corrected volume was computed (C_vol_) by dividing the volume by TIV. Furthermore, for each slice, the contrast-to-noise ratio (CNR) was computed by normalizing the mean signal in the SNc relative to the signal in the background similar to our previous studies^25,31^ as follows:

$${\text{CNR}} = {\text{Mean}}\_{\text{over}}\_{\text{slices}}\left\{ {\left( {{\text{Sig}}_{{{\text{SNc}}}} - {\text{Sig}}_{{{\text{BND}}}} } \right)/{\text{STD}}_{{{\text{BND}}}} } \right\}$$

where Sig_SNc_ is the signal intensity in the SNc ROI, Sig_BND_ the signal intensity in background ROI and STD_BND_ the standard deviation in background ROI.

##### Regional SNc

The final regional SNc masks in form of the intersections as explained above were used for computing the volumes and CNR separately for the sensorimotor, associative, and limbic regions for each participants. Similar to the whole SNc volume as explained above, the regional SNc volumes were also calculated using in-house MATLAB algorithm by employing fslstats function of FSL as the number of voxels in the intersectional location. CNR was computed as the ratio between signal in the regional ROIs and the signal in the background ROI using the same formula as the whole SNc.

Lastly, an average of the left and right SNc was used for further statistical analyses for both whole and regional SNc.

### Statistical analyses

All analyses were performed using R (R Core Team 2019, v3.6.1). A one-way analysis of variance (ANOVA) was performed to compare the demographic and clinical characteristics between the three groups. Chi-square test was used for sex proportion. An analysis of covariance (ANCOVA) was performed to explore the differences of whole and regional NM-MRI values among the groups while controlling the effect of age and sex. Post-hoc student's t-tests were also performed when group effects using ANCOVA were significant. The *p*-values below 0.05 were considered to be statistically significant at the global level. The diagnostic value was calculated using receiver operator characteristic (ROC). The correlations between the NM-MRI measurements and clinical scores were also obtained by performing univariate Pearson correlations using a permutation method to control for family-wise error rate^34^. Furthermore, an independent-samples t-test was also acquired to assess the difference between the G2385R and R1628P mutation carriers.

### Ethical approval

This experiment was performed in accordance with the Declaration of Helsinki and was approved by the Institutional Review Board of Xuanwu Hospital. All subjects provided written informed consent prior to the experiment.

## Results

The demographic and clinical characteristics of the subjects are shown in Table 1. No significant differences were observed in age and sex proportion among the three groups (χ^2^ = 1.728, *p* = 0.421). There were significant differences between the groups in MDS-UPDRS scores. Furthermore, MDS-UPDRS scores in PD patients were significantly higher compared to HCs and asymptomatic LRRK2 carriers.

**Table 1:** Demographic and clinical characteristics of the three groups.

	HCs	Asymptomatic LRRK2 Carriers	PD	POST-HOC	POST-HOC	POST-HOC
				HCs vs LRRK2	HCs vs PD	LRRK2 vs PD
				*p*-value	*p*-value	*p*-value
Age (Years)	64.4 ± 4.2	67.1 ± 4.8	64.2 ± 6.4	*0.098****#***	0.856	*0.087****#***
Sex (M/F)	16/14	10/5	11/11			
Duration (Years)	–	–	5.25 ± 4.28	–	–	–
MDS-UPDRS I	2.23 ± 3.15	3.33 ± 3.29	10.73 ± 7.26	0.482	< 0.001*	< 0.001*
MDS-UPDRS II	0.13 ± 0.43	0.93 ± 1.87	11.82 ± 5.79	0.465	< 0.001*	< 0.001*
MDS-UPDRS III	1.53 ± 2.42	1.60 ± 2.10	30.77 ± 11.38	0.975	< 0.001*	< 0.001*
MDS-UPDRS IV	–	–	0.64 ± 1.87	–	–	–
H&Y scale	–	–	2.09 ± 0.65	–	–	–
LEDD (mg)	–	–	524.4 ± 301.2	–	–	–

F: female; HCs: healthy controls; H & Y: Hoehn-Yahr disability scale; LEDD: Levodopa equivalent daily doses; LRRK2: leucine-rich repeat kinase 2; M: male; PD: Parkinson’s disease; MDS-UPDRS: Movement disorder society Unified Parkinson’s Disease Rating Scale. Post-hoc analyses were performed with Fisher's least significant difference corrected for multiple comparisons. * indicates significant *p* values < 0.05 and # indicates trending *p* values between 0.05 and 0.09; “–”: not applicable. Values represented as mean ± standard deviation.

There was a high reproducibility of the SNc measurements performed by both the raters with an excellent Dice coefficient between the segmentations (intra-rater 1: 0.87 and rater 2: 0.81, inter-rater: 0.81). The ICC values were high for intra-rater 1 (ICC for whole SNc volume: 0.82) and intra-rater 2 (ICC for CNR: 0.79).

**Figure 2:**
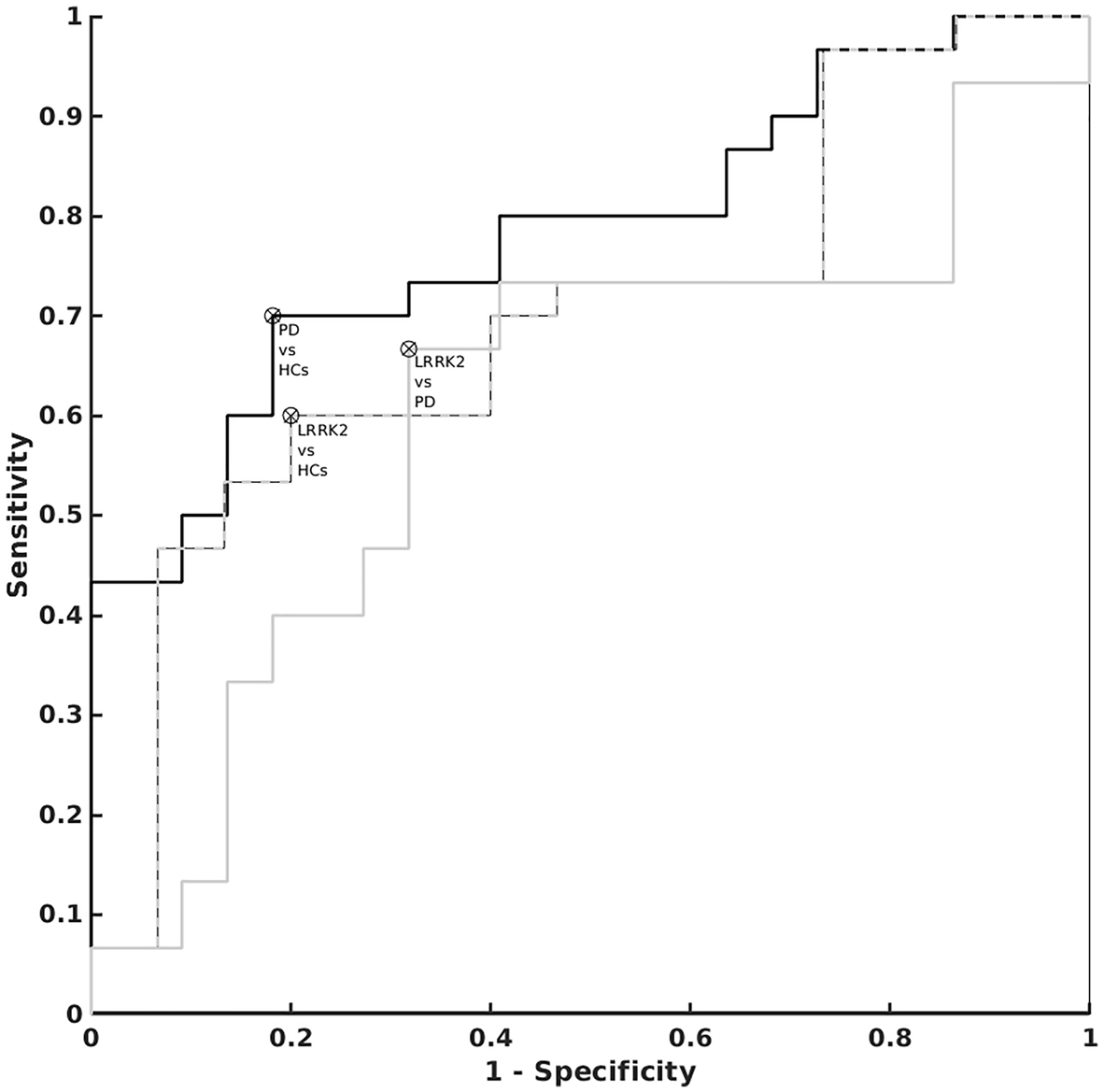
The area under the ROC curves for contrast to noise ratio (CNR) in the whole SNc of 0.78 between PD patients and HCs, 0.70 between asymptomatic LRRK2 carriers and HCs and 0.61 between asymptomatic LRRK2 carriers and PD patients.

The ROC analyses provided areas under the curve of 0.66 for whole SNc volume, 0.61 for C_vol_, and 0.70 for CNR between HCs and LRRK2, 0.79 for Vol, 0.75 for C_vol_, and 0.78 for CNR between HCs and PD, and 0.66 for Vol, 0.61 for C_vol_, and 0.61 for CNR between LRRK2 and PD (Fig. 2).

The NM-MRI measurements in whole and regional SNc are shown in Table 2.

**Table 2:** Neuromelanin-sensitive MRI measurements in whole and regional substantia nigra pars compacta (SNc).

		HCs	Asymptomatic LRRK2 Carriers	PD	ANCOVA	POST-HOC		
						HCs vs LRRK2	HCs vs PD	LRRK2 vs PD
Whole SNc								
	Volume (mm^3^)	394.2 ± 57.4	359.4 ± 62.6	324.6 ± 60.8	< 0.001*	*0.069*^*#*^	< 0.001*	0.100
	C_vol_	0.27 ± 0.04	0.24 ± 0.05	0.23 ± 0.04	0.004*	0.111	< 0.001*	0.290
	CNR	1.51 ± 0.19	1.38 ± 0.19	1.32 ± 0.13	< 0.001*	0.043*	< 0.001*	0.261
Regional SNc								
Limbic	Volume (mm^3^)	85.1 ± 11.6	75.9 ± 13.8	82.8 ± 14.2	0.104	**–**	–	–
	CNR	1.58 ± 0.26	1.54 ± 0.23	1.38 ± 0.32	0.028*	0.616	0.015*	0.119
Associative	Volume (mm^3^)	82.8 ± 12.3	76.7 ± 14.2	71.9 ± 12.6	0.009*	0.162	0.004*	0.314
	CNR	1.83 ± 0.29	1.74 ± 0.43	1.58 ± 0.31	0.044*	0.459	0.007*	0.221
Sensorimotor	Volume (mm^3^)	74.6 ± 16.3	73.0 ± 16.8	64.0 ± 14.7	*0.069*^*#*^	0.776	0.022*	0.108
	CNR	0.94 ± 0.20	0.92 ± 0.16	0.76 ± 0.30	0.015*	0.843	0.016*	*0.082*^*#*^

ANCOVA, Analysis of covariance; CNR, Contrast to Noise Ratio; C_vol_, Corrected volume; HCs, healthy controls; LRRK2, Leucine-rich repeat kinase 2; PD, Parkinson’s disease; “–”, Not applicable.

Post-hoc analyses were performed using T-test. * indicates significant *p* values < 0.05 and # indicates trending *p* values between 0.05 and 0.09. Values represented as mean ± standard deviation.

### Whole SNc

**Figure 3:**
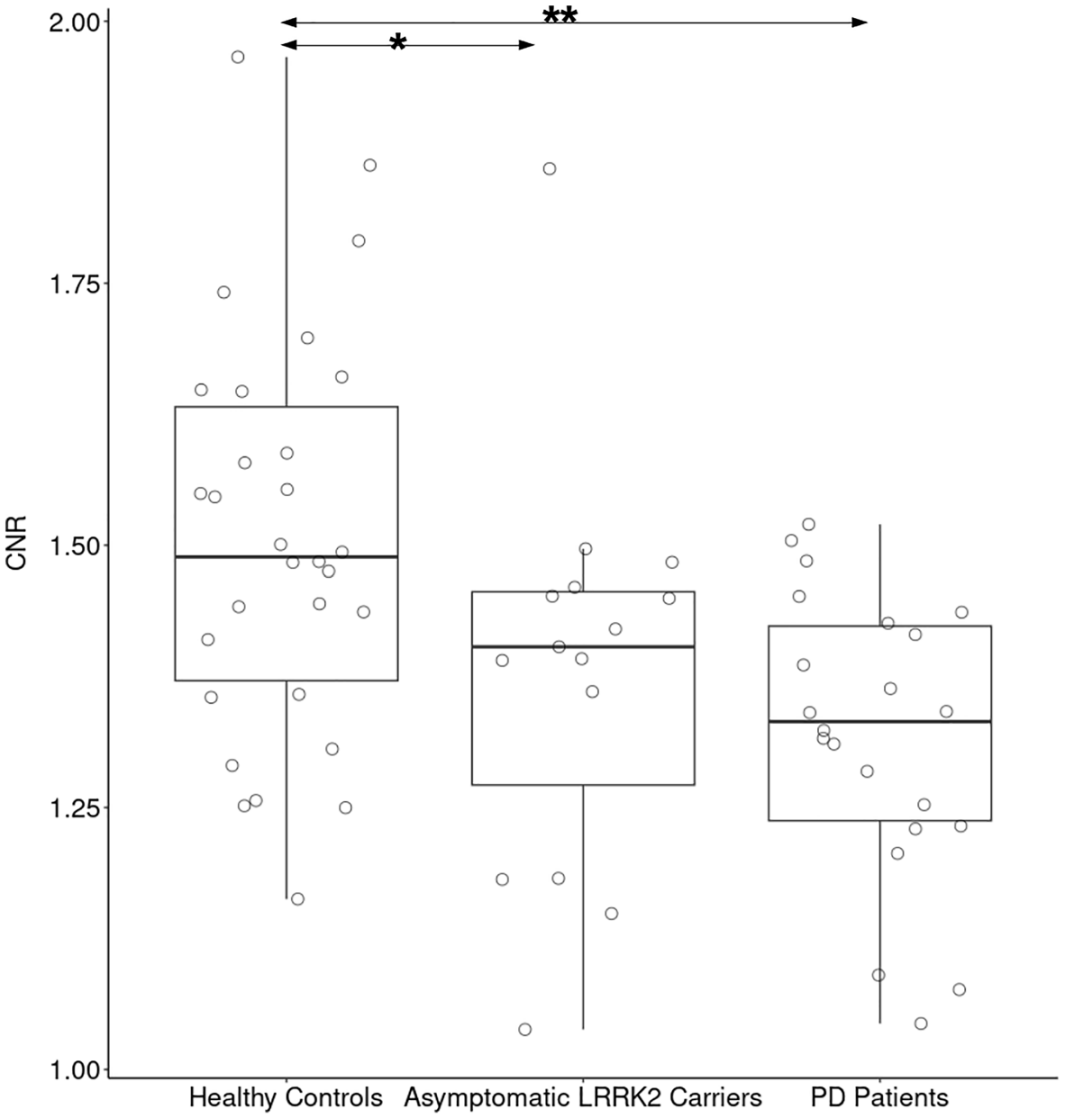
Box plot of contrast to noise ratio (CNR) in the whole SNc of the three groups. Post-hoc analyses were performed using T-test. * indicates significant *p* values < 0.05 and  ** indicates *p* values  < 0.001.

There were highly significant group effects for all SNc measurements (volume, C_vol_ and CNR). Between HCs and LRRK2, there was a significant difference in CNR (Fig. 3) with a percentage change of − 8.4%, a trending difference of − 8.8% for volume and a non-significant difference of − 9.2% for C_vol_. Compared to HCs, PD had highly significant percentage changes of − 17.7% in volume, − 15.7% in C_vol_, and − 12.5% for CNR. Between LRRK2 and PD, there was no significant difference in any whole SNc measurements.

No significant differences were found in the NM-MRI values in the whole SNc values between G2385R and R1628P mutation carriers using Post-hoc student's t-tests (for volume, *p* = 0.40, for C_vol_, *p* = 0.17 and for CNR, *p* = 0.16).

### Regional SNc

There were significant group effects for CNR in all SNc regions and for volumes in the associative region, with a trend for the sensorimotor region and no significant changes for the limbic region. Hence, Post-hoc analyses were only performed when group effects were significant. Post-hoc analyses did not show any significant change in CNR between HCs and LRRK2 in any region or in volume in the associative and sensorimotor regions.

Between the HCs and PD, the sensorimotor region showed the greatest changes in all measures with decreases of − 14.2% for volume, and − 19.0% for CNR. The associative region showed significant decreases of − 12.9% for volume, and − 15.9% for CNR while the limbic region showed significant changes only for CNR (− 13.5%) along with a non-significant − 2.3% volume decrease.

Between LRRK2 and PD, only the sensorimotor region demonstrated a trend for CNR.

### Correlation analysis

The correlations between imaging measures and clinical variables are shown in Table 3. Asymptomatic LRRK2 carriers showed significant negative correlations between MDS-UPDRS-I scores and whole SNc volume, and between MDS-UPDRS-II and both volume and C_vol_ separately. PD patients showed significant negative correlations between MDS-UPDRS-I and CNR.

**Table 3:** Correlations between imaging measures and clinical variables using a multiple comparison permutation method.

Whole SNc	Volume (mm^3^)	C_vol_	CNR			
	*p*	r	*p*	r	*p*	r
Healthy Controls						
MDS-UPDRS-III OFF	0.304	0.089	0.379	0.047	**0.022**	− 0.350
Asymptomatic LRRK2 Carriers						
MDS-UPDRS-I OFF	**0.010**	− 0.596	0.106	− 0.355	0.210	− 0.225
MDS-UPDRS-II OFF	**0.048**	− 0.444	**0.032**	− 0.479	0.149	0.223
MDS-UPDRS-III OFF	0.256	− 0.177	**0.032**	− 0.529	0.244	0.177
Patients with PD						
MDS-UPDRS-I OFF	0.271	− 0.123	0.316	− 0.102	**0.034**	− 0.423
MDS-UPDRS-II OFF	0.310	− 0.091	0.249	− 0.153	0.249	− 0.201
MDS-UPDRS-III OFF	**0.012**	− 0.474	**0.011**	− 0.473	0.311	0.110
MDS-UPDRS-IV OFF	0.402	− 0.033	0.515	0.039	0.106	0.265
H&Y score	0.469	0.020	0.423	0.044	0.194	− 0.180
Disease Duration	0.226	− 0.157	0.277	− 0.127	0.323	− 0.093
Levodopa Equivalent Dose	0.336	0.107	0.220	0.174	0.386	0.065

CNR, Contrast to Noise Ratio; C_vol_, Corrected volume; H&Y score, Hoehn and Yahr scale; HCs, Healthy controls; LRRK2, leucine-rich repeat kinase 2; PD, Parkinson’s disease; MDS-UPDRS, Movement Disorder Society Unified Parkinson’s Disease Rating Scale.

*indicates significant *p* values < 0.05 and # indicates trending *p* values between 0.05 and 0.09.

**Figure 4:**
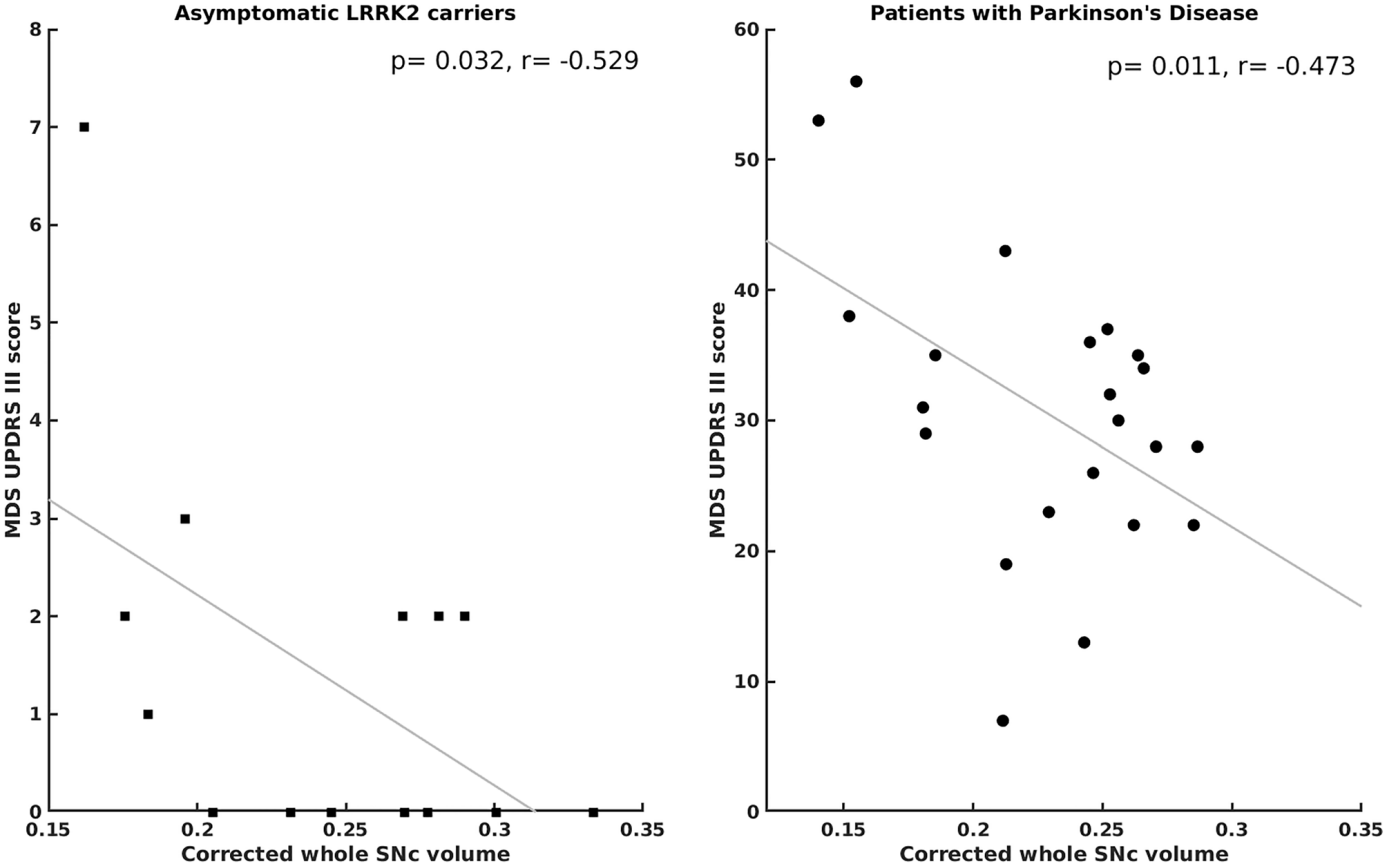
Scatter plot between MDS-UPDRS-III scores and corrected whole SNc volume in LRRK2 and PD patients demonstrating significantly negative correlations.

Asymptomatic LRRK2 carriers and PD patients showed significant negative correlations between MDS-UPDRS-III scores and C_vol_ (Fig. 4), whereas the HCs showed significant negative correlations between MDS-UPDRS-III scores and CNR.

We did not observe any correlations between disease duration or H&Y score and any whole SNc measure. Similarly, no correlations were observed between Levodopa Equivalent Daily Doses (LEDD in mg) of PD patients and any whole SNc measure.

## Discussion

We employed NM-MRI to investigate global and regional nigral NM changes in asymptomatic LRRK2 carriers. The main findings were that we observed a decrease in CNR in the whole SNc in asymptomatic LRRK2 carriers and PD patients compared with the HCs. PD patients also had lower whole SNc volumes compared with the HCs. We found correlations between the MDS-UPDRS III scores and the whole SNc C_vol_ in asymptomatic LRRK2 carriers and PD patients.

The reduced CNR in asymptomatic LRRK2 carriers compared with the HCs indicated that the degeneration of dopaminergic neurons already existed in the SN in the preclinical stage of the disease. This was in line with previous transcranial sonography or PET studies showing nigrostriatal impairment in asymptomatic LRRK2 carriers^12^. Interestingly, there was no significant difference in SNc volume or CNR between asymptomatic LRRK2 carriers and PD patients. An imaging study also found that iron deposition in the SN in asymptomatic LRRK2 carriers was at the same level as in patients with PD^16^. These results suggest that asymptomatic carriers of LRRK2 already exhibit nigral neurodegeneration.

Asymptomatic LRRK2 carriers had decreased CNR compared to HCs with a trend for volume, and higher CNR and volume compared to PD patients although not significant. The volume and CNR values in asymptomatic LRRK2 carriers were thus intermediate between those in the two other groups, smaller than that in HCs and larger than that in PD patients.

Asymptomatic LRRK2 carriers did not reach a significant level compared to idiopathic PD patients, regardless of the imaging variable. This could likely be because the threshold for symptom onset was not reached in these asymptomatic LRRK2 carriers. This could also be due to compensatory mechanisms suggesting that the remaining dopaminergic neurons were able to maintain sufficient nigrostriatal dopaminergic function. Compensatory mechanisms such as increased dopamine turnover^35^, elevated serotonin transporter binding^13^, and increased functional connectivity between the ventroanterior putamen have also been reported in asymptomatic LRRK2 mutation carriers^36^. These effects may compensate for nigrostriatal damage and help maintain relatively normal functions in the preclinical phase of genetic PD^35^. Further studies are warranted to understand the conversion of genetic mutation carriers from an asymptomatic to a symptomatic state.

The negative correlations between MDS-UPDRS-I and II and whole SNc volume, and MDS-UPDRS-III scores and C_vol_ in asymptomatic LRRK2 carriers suggested that the nigral NM signal decrease was potentially associated with the early sub-threshold appearance of both non-motor and motor symptoms. However, further longitudinal NM-MRI studies are warranted to investigate the relationship between nigral NM changes and the appearance of such symptoms in parkinsonism particularly to examine the utility of NM-MRI in predicting PD onset. The negative correlation between C_vol_ and MDS-UPDRS-III scores in PD patients was in line with our previous studies^24,25,28^. Interestingly, we also observed significant negative correlations between MDS-UPDRS-III scores and CNR in the HCs. These decreasing CNR values likely reflected the presence of subthreshold degeneration of SNc dopaminergic neurons in these healthy individuals^37,38^ responsible for a mild increase in MDS-UPDRS-III scores.

The lack of correlations between disease duration and NM-MRI measures in PD could be due to the moderate stage of patients in this study (5.25 ± 4.28 years). Moreover, we did not observe any correlations between LEDD intake of patients and NM-MRI measures. This was in line with previous studies^24,25^ suggesting that NM signal changes were not influenced by the dopaminergic medication of the patients.

Consistent with previous NM-MRI studies^19,22,24,25,39^, PD patients demonstrated a decrease in volume and CNR in the whole SNc. Further, PD patients also showed a decrease in CNR in the regional SNc in line with our previous studies^31,32^ suggesting the predominant involvement of the posterolateral nigral territory that corresponds to the sensorimotor region. The NM-MRI values in the SNc had a moderate accuracy to discriminate asymptomatic LRRK2 mutation carriers from HCs, while the combination of CNR and volume might increase the discriminating power. As not all LRRK2 mutation carriers will develop PD in their lifetime, nigral degeneration in some of them may not be significant. In contrast, the NM-MRI values in the SNc had a high accuracy to discriminate PD patients from HCs for whole SNc volumes similar to previous studies^27,41^.

Using whole SNc measures, we did not find differences between LRRK2 G2385R and R1628P mutation carriers, which suggests that these two mutations have similar pattern of overall nigral degeneration. However, as the sample size was small, this finding needs to be verified in a larger cohort.

Our study had a few limitations. Firstly, the sample size was relatively small, thus, larger cohorts are needed to validate our findings. Secondly, longitudinal studies are needed to verify whether global and regional nigral NM changes can predict PD conversions. Thirdly, although CNR showed significant group differences in both regional and whole SNc, the regional volumes were not always significant. This could be due to an artificial ceiling effect created by the usage of the template if the region that was manually delineated was bigger than the template. Fourthly, although we used automatic template-based segmentation for regional SNc ROIs, we used manual segmentation for the whole SNc ROIs. Automated methods may improve the reproducibility of segmentation techniques^25,28,41,42^. However, experienced raters can achieve excellent reproducibility of manual measurements as our rater 1 achieved intra-rater ICC volume of 0.82 in line with several studies (ICC ranging 0.69–0.95 for manual measurements)^42–46^. Recent NM-MRI studies demonstrated nigral NM damage in LRRK2-associated PD patients^47,48^. Although similar studies validated in several cohorts will help better understand whether nigral neurodegeneration mechanism is similar in genetic and idiopathic PD.

In conclusion, using the NM-MRI technique, we demonstrated globally decreased volume and signal intensity in the SNc in asymptomatic LRRK2 carriers suggesting early nigral neurodegeneration in these subjects. Longitudinal studies involving larger sample sizes could help determine whether NM-MRI can detect asymptomatic LRRK2 carriers that are at risk of developing overt parkinsonism.

## Data Availability

The data that support the findings of this study are available on request from the corresponding author [TW]. The data are not publicly available due to them containing information that could compromise research participant privacy/consent.

## Code availability

The code or algorithm can be accessed on reasonable request from the corresponding authors [SL, RG].
